# Contraceptive Method Provision Patterns Among Rural and Urban Kentucky Medicaid Enrollees

**DOI:** 10.1111/jrh.70160

**Published:** 2026-05-04

**Authors:** Dustin K. Miracle, Svetla Slavova, Jeffery Talbert, Daniela C. Moga, Patricia R. Freeman

**Affiliations:** ^1^ Department of Pharmacy Practice and Science University of Kentucky College of Pharmacy Lexington Kentucky USA; ^2^ Department of Biostatistics University of Kentucky Lexington Kentucky USA; ^3^ Institute for Biomedical Informatics University of Kentucky Lexington Kentucky USA; ^4^ Department of Epidemiology University of Kentucky College of Public Health Lexington Kentucky USA; ^5^ Sanders‐Brown Center on Aging University of Kentucky Lexington Kentucky USA; ^6^ Center for the Advancement of Pharmacy Practice University of Kentucky College of Pharmacy Lexington Kentucky USA

**Keywords:** contraception, contraception access, long‐acting reversible contraception, rural health, women's health

## Abstract

**Purpose:**

To characterize contraceptive provision among the Kentucky Medicaid population and assess for rural−urban disparities.

**Methods:**

Kentucky Medicaid claims from the calendar year 2019 were used to identify females at risk for unintended pregnancy (via a modified version of the criteria defined by the US Department of Health and Human Services Office of Population Affairs). Multinomial logistic regression was used to assess the impact of rural−urban residence on contraceptive outcomes, while adjusting for relevant covariates. Outcomes for the multinomial regression model were provision of a: (1) less effective method (i.e., condoms) or no contraceptive; (2) moderately effective method (oral, transdermal, injectable, or vaginal); (3) or long‐acting reversible contraceptive (LARC) method.

**Findings:**

A total of 239,160 enrollees at risk for unintended pregnancy were included for analyses. Adjusted odds of provision of a moderately effective method (vs. a less effective method or no method) were higher among both those residing in rural‐adjacent to urban (aOR 1.17; 95% CI, 1.13–1.20) and rural‐nonadjacent to urban (aOR 1.15; 95% CI, 1.12–1.18) locations compared to urban. Notably, adjusted odds of provision of an LARC method (vs. a less effective method or no method) were significantly lower among those residing in rural‐nonadjacent to urban locations (aOR 0.81; 95% CI 0.77–0.85) compared to those in urban locations.

**Conclusions:**

Despite high moderately effective contraceptive provision among Kentucky Medicaid enrollees in rural‐nonadjacent to urban counties, adjusted odds of LARC provision are significantly lower, signaling significant barriers to access among this population.

## 1 Introduction

In the United States, despite modest reductions in recent years, nearly half of pregnancies remain unintended [1]. Highlighting the growing focus on addressing this issue, the US Department of Health and Human Services’ (DHHS) Healthy People 2030 goals include several objectives aimed at increasing contraception use and decreasing unintended pregnancies [2]. While work conducted using data from the National Survey of Family Growth (NSFG) and the Behavioral Risk Factor Surveillance System (BRFSS) has indicated little growth among contraceptive use rates and high variation among states, little work has been conducted utilizing administrative claims data [2, 3].

Prior studies have detailed existing disparities in contraceptive use rates, including among Black and Hispanic individuals [4, 5, 6, 7, 8] and individuals with opioid use disorder [9, 10, 11, 12]; however, data are less conclusive regarding the impact of rural residence on contraceptive access, with conflicting results from survey data and sparse administrative claims‐based work [13, 14, 15, 16, 17]. Kentucky is largely rural, with 85 of Kentucky's 120 counties being classified by Rural‐Urban Continuum Codes (RUCCs) as such [18]. Healthcare access, including contraceptives, in these rural areas may be limited due to a shortage of healthcare providers; of Kentucky's 262 Health Professional Shortage Areas, 87% are rural [19]. Further, recent estimates indicate that over 100,000 women live in Kentucky counties without a single provider able to provide a full range of contraceptive methods—many of which are rural [20].

Although existing literature has characterized both state‐level contraceptive use rates and Medicaid‐specific state‐level rates, studies have excluded individuals from Kentucky, due to suspected data quality issues with Medicaid‐specific rates in prior years and the fact that Kentucky does not field the BRFSS Family Planning section [3, 21]. Kentucky was one of the first US states to expand its Medicaid program under the Affordable Care Act, expanding eligibility in 2014 to include nearly all adults with incomes up to 138% of the federal poverty level [22]. As such, Kentucky has an expansive Medicaid program, encompassing approximately 1.2 million enrollees in 2019 and providing health coverage for one in four Kentuckians [23, 24]. In 2023, Kentucky was one of seven US states whose Medicaid program provided health coverage for over 25% of the state's population [25]. Notably, over half of Kentucky Medicaid enrollees live in rural areas [25]. Given the lack of data regarding contraceptive use rates among Kentucky Medicaid enrollees, this study sheds light on issues faced within a large, unstudied population and further examines questions relative to contraceptive access for those in rural areas.

The primary objective of this study was to characterize contraceptive provision among the Kentucky Medicaid population and assess for rural−urban disparities. Secondary objectives include exploring provider‐related differences in the method of contraceptives provided and variations in provider type by rural−urban classification of the contraceptive recipient.

## 2 Methods

### 2.1 Sampling

This cross‐sectional study was conducted using Kentucky Medicaid claims from the calendar year 2019 (allowing for an assessment of the typical Kentucky Medicaid population prior to the large influx of patients during the COVID‐19 pandemic). Enrollees at risk of unintended pregnancy were identified via a modified version of the DHHS Office of Population Affairs (OPA) guidelines for evaluating contraceptive care [26]. Subjects included were females aged 15−44 (as of December 31, 2019) with a primary residence within Kentucky and continuous enrollment with no more than the allowable gap (45 days) for the calendar year 2019. To determine continuous enrollment for a beneficiary for whom enrollment is verified monthly, the beneficiary must not have had more than a 1‐month gap in coverage. Exclusion criteria included individuals with dual enrollment, those infecund due to noncontraceptive reasons, those with live birth within the last 2 months of the calendar year, and those still pregnant at the end of the calendar year. All inclusion and exclusion criteria were applied by the Kentucky Cabinet for Health and Family Services (CHFS) Office of Data Analytics (ODA) prior to the provision of requested data per data use agreement terms. While DHHS OPA definitions include no lookback period to exclude those with previously placed long‐acting reversible contraceptive (LARC) methods, a 5‐year lookback period was used to exclude individuals with prior LARC placement and no subsequent removal prior to the calendar year 2019. A sensitivity analysis was conducted using DHHS OPA definitions to assess for additional benefit when using the lookback period.

### 2.2 Definitions

Prescription and medical claims for contraceptive provision were identified via National Drug Codes (NDCs), International Classification of Diseases – 10th Revision Clinical Modification (ICD‐10‐CM) codes, Current Procedural Terminology (CPT) codes, and Healthcare Common Procedure Coding System (HCPCS) codes provided in DHHS OPA contraceptive code lookup tables [27]. For subjects who received more than one contraceptive method during the calendar year, the most recent method was used for analysis. Preventative health visits were identified via ICD‐10‐CM codes utilized in existing literature [28]. Enrollees with opioid use disorder (OUD) were identified via ICD‐10‐CM, CPT, and HCPCS codes using the National Quality Forum (NQF) approach, requiring at least one ICD‐10‐CM code and at least one CPT/HCPS code during the study timeframe [29]. Pregnancy was identified using ICD‐10‐CM, CPT, and HCPDS Codes listed in DHHS OPA lookup tables, as well as labor and delivery records (i.e., birth certificate records held by Kentucky CHFS Office of Vital Statistics). A sensitivity analysis was conducted to assess the added benefit of the additional labor and delivery record criteria as compared to the DHHS OPA methodology. All codes not listed in the DHHS OPA lookup tables are listed in Appendix S1. Rural−urban classification was determined via 2023 RUCCs linked to enrollee county of residence, with “urban” corresponding to metropolitan areas (RUCCs 1−3), “rural‐adjacent to urban” corresponding to nonmetropolitan areas adjacent to metropolitan areas (RUCCs 4, 6, and 8), and “rural‐nonadjacent to urban” corresponding to nonmetropolitan areas not adjacent to metropolitan areas (RUCCs 5, 7, and 9) [30]. Age was categorized into two groups per DHHS OPA reporting guidelines: ages 15−20 and 21−44. Provider type is reported as categorized by Kentucky CHFS.

### 2.3 Statistical Analysis

Descriptive analyses included calculation of the percentage of female enrollees ages 15−44 who were provided (1) an LARC method (intrauterine devices (IUD/systems or subdermal implants); or (2) a moderately effective formulation (oral, transdermal, injectable, or vaginal) [31]. For both measures, the denominator value was the number of Kentucky Medicaid enrollees between ages 15−44 at risk for unintended pregnancy. These measures were calculated and stratified by rural−urban residency along with other demographic factors, including age, race/ethnicity (as reported to Medicaid), OUD diagnosis, Medicaid type (traditional vs. Affordable Care Act expansion), and preventative health visit during the study period. Medicaid type was included in the analysis as a surrogate for income data, which were not made available to researchers. Sensitivity analyses were conducted, defining pregnancy without use of labor and delivery records and not utilizing a 5‐year lookback period to exclude those with previous LARC placement.

A multinomial logistic regression analysis was conducted to identify any associations between rural−urban residence and contraceptive outcome, while adjusting for relevant covariates (age, race/ethnicity, preventive healthcare visit, traditional/expansion Medicaid, opioid use disorder). Outcomes for the multinomial regression model were classified using established efficacy definitions as provision of a: (1) less effective method (i.e., condoms) or no contraceptive (reference); (2) moderately effective method; (3) or LARC method [31]. Adjusted odds ratios (aORs) and 95% confidence intervals (CIs) are reported for rural−urban classifications as well as each covariate included in the model. Statistical analyses were conducted via RStudio version 2023.09.1 (Posit Software PBC). Choropleth maps were developed using QGIS version 3.22.16 (Open Source) to display variations in contraceptive provision across Kentucky counties.

The institutional review board at the University of Kentucky approved the study protocol.

## 3 Results

### 3.1 Sample

A total of 239,160 enrollees at risk for unintended pregnancy were included for analyses. Most were between ages 21−44 (74.4%), were White (73.7%), and held residence in urban locations (52.8%) (Table 1). Roughly one‐fourth (26.2%) received a preventive healthcare visit within the calendar year, and a few (1.2%) were diagnosed with OUD.

**TABLE 1:** Individuals at risk for unintended pregnancy and contraceptive provision among Kentucky Medicaid enrollees, 2019 (*N*=239,160).

Demographic	Individuals at risk for unintended pregnancy, *n* (%^a^)	Individuals provided a moderately effective method of contraception^c^, *n* (%^b^)	Individuals provided an LARC method^d^, *n* (%^b^)
Overall	239,160 (100.0)	54,810 (22.9)	10,602 (4.4)
Rural−urban residency classification			
Urban	126,188 (52.8)	27,368 (21.7)	6046 (4.8)
Rural‐adjacent to urban	46,856 (19.6)	11,447 (24.4)	2126 (4.5)
Rural‐nonadjacent to urban	66,116 (27.6)	15,995 (24.2)	2430 (3.7)
Age			
15−20	61,277 (25.6)	21,386 (34.9)	2974 (4.9)
21−44	177,883 (74.4)	33,424 (18.8)	7628 (4.3)
Diagnosis of opioid use disorder			
No	236,367 (98.8)	54,439 (23.0)	10,487 (4.4)
Yes	2793 (1.2)	371 (13.3)	115 (4.1)
Preventive healthcare visit			
No	176,445 (73.8)	32,639 (18.5)	5595 (3.2)
Yes	62,715 (26.2)	22,171 (35.4)	5007 (8.0)
Race/ethnicity			
Black	29,797 (12.5)	6258 (21.0)	1372 (4.6)
Hispanic	6530 (2.7)	1319 (20.2)	379 (5.8)
White	176,366 (73.7)	41,543 (23.6)	7823 (4.4)
Other	26,467 (11.1)	5690 (21.5)	1028 (3.9)
Medicaid qualification			
Traditional	110,887 (46.4)	26,906 (24.3)	4847 (4.4)
Expansion	128,273 (53.6)	27,904 (21.8)	5755 (4.5)

Abbreviation: LARC, long‐acting reversible contraceptive method.

^a^ Column percentages.

^b^ Row percentages.

^c^ Moderately effective contraceptive methods included oral, transdermal, vaginal, and injectable methods.

^d^ LARC methods included were intrauterine devices/systems and subdermal implants.

### 3.2 Descriptive Measures

**FIGURE 1:**
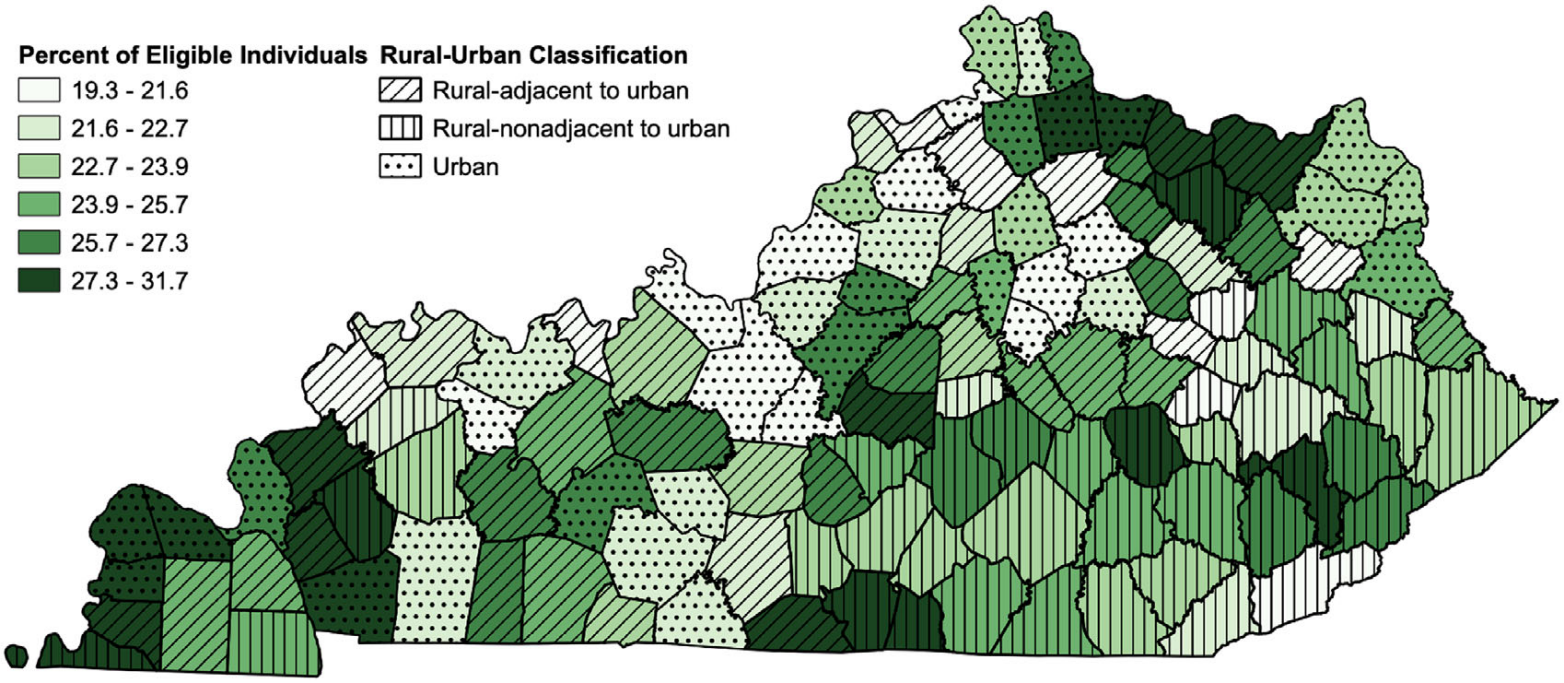
Percentage of female Kentucky Medicaid enrollees at risk of unintended pregnancy provided a moderately effective method of contraception, 2019.

**FIGURE 2:**
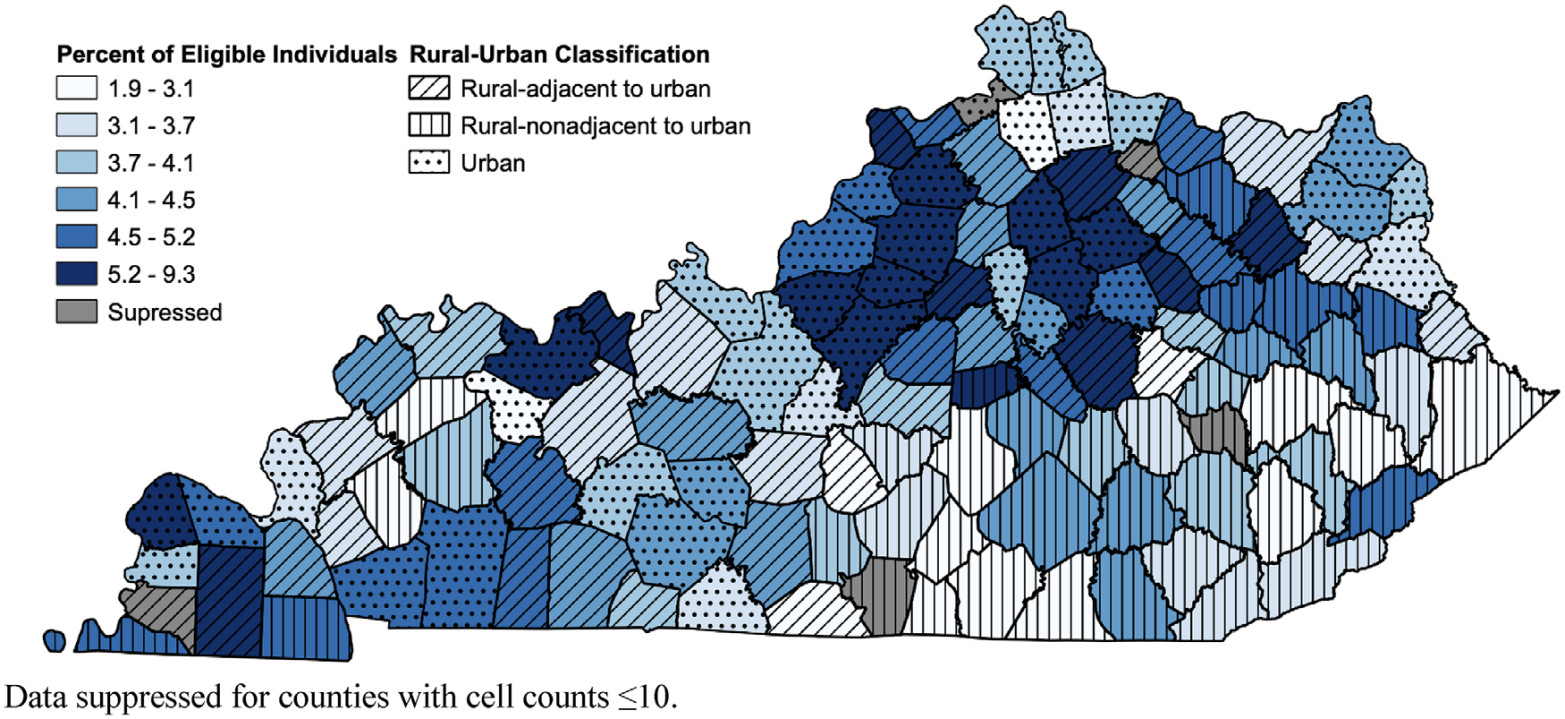
Percentage of female Kentucky Medicaid enrollees at risk of unintended pregnancy provided a long‐acting reversible method of contraception (LARC), 2019.

The percentage of female Kentucky Medicaid enrollees aged 15−44 who were provided a moderately effective formulation (oral, transdermal, injectable, vaginal) was 22.9%. This measure was highest among those living in rural‐adjacent to urban areas (24.4%) and lowest among those in urban areas (21.7%). The percentage of enrollees provided an LARC method (intrauterine devices/systems or subdermal implants) was 4.4%. This measure, however, was highest among those in urban areas (4.8%) and notably lowest among those living in rural‐nonadjacent to urban areas (3.7%). Figure 1 illustrates the geographic variation in the provision of moderately effective formulations among Kentucky Medicaid enrollees. Geographic variations regarding the provision of an LARC formulation are displayed in Figure 2.

### 3.3 Factors Associated With Odds of Contraceptive Provision

Results from the multinomial regression model are summarized in Table 2. Compared to enrollees residing in urban locations, higher adjusted odds of provision of a moderately effective method (vs. a less effective method or no method) were observed among both those residing in rural‐adjacent to urban (aOR 1.17; 95% CI, 1.13–1.20) and rural‐nonadjacent to urban (aOR 1.15; 95% CI, 1.12–1.18) locations. Conversely, adjusted odds of provision of an LARC method (vs. a less effective method or no method) were significantly lower among those residing in rural‐nonadjacent to urban locations (aOR 0.81; 95% CI 0.77–0.85) compared to those in urban locations. No significant differences in the adjusted odds of LARC provision (vs. a less effective method or no method) were observed among rural‐adjacent to urban (aOR 1.00; 95% CI 0.95–1.06) compared to urban.

**TABLE 2:** Results of multinomial logistic regression model assessing odds of being provided a moderately effective method or long‐acting reversible contraceptive method (vs. less effective method or no contraceptive) among Kentucky Medicaid enrollees, 2019 (*N*=239,160).

	Moderately effective method^a^ (vs. less effective or no contraceptive method)	*p*‐value	LARC method^b^ (vs. less effective or no contraceptive method)	*p*‐value
Variable	aOR (95% CI)		aOR (95% CI)	
Rural−urban classification				
Urban	Reference	Reference	Reference	Reference
Rural‐adjacent to urban	1.17 (1.13–1.20)	<0.001	1.00 (0.95–1.06)	0.957
Rural‐nonadjacent to urban	1.15 (1.12–1.18)	<0.001	0.81 (0.77–0.85)	<0.001
Age				
15−20	Reference	Reference	Reference	Reference
21−44	0.29 (0.28–0.29)	<0.001	0.47 (0.45–0.50)	<0.001
Diagnosis of opioid use disorder				
No	Reference	Reference	Reference	Reference
Yes	0.58 (0.52–0.65)	<0.001	0.82 (0.68–0.99)^a^	0.041
Preventive healthcare visit				
No	Reference	Reference	Reference	Reference
Yes	3.71 (3.62–3.79)	<0.001	4.24 (4.07–4.42)	<0.001
Race/ethnicity				
White	Reference	Reference	Reference	Reference
Black	0.86 (0.84–0.89)	<0.001	0.91 (0.85–0.97)	0.002
Hispanic	0.69 (0.64–0.73)	<0.001	1.05 (0.94–1.18)	0.352
Other	0.86 (0.83–0.89)	<0.001	0.80 (0.74–0.86)	<0.001
Medicaid qualification				
Traditional	Reference	Reference	Reference	Reference
Expansion	1.19 (1.16–1.21)	<0.001	1.08 (1.03–1.13)	<0.001

*Note*: Estimates are from a multinomial logistic regression model, with reference outcome being a less effective method of contraception (i.e., condoms, withdrawal, calendar) or no method of contraception.

Abbreviations: aOR, adjusted odds ratio; CI, confidence interval; LARC, long‐acting reversible contraceptive method.

^a^ Moderately effective contraceptive methods included oral, transdermal, vaginal, and injectable methods.

^b^ LARC methods included were intrauterine devices/systems and subdermal implants.

Those aged 21−44 were at lower adjusted odds of being provided either a moderately effective method (aOR 0.29; 95% CI, 0.28–0.29) or LARC method (aOR 0.47; 95% CI, 0.45–0.50) (vs. a less effective method or no method) compared to those aged 15−20. Notably, adjusted odds of being provided either a moderately effective method (aOR 0.58; 95% CI, 0.52–0.65) or an LARC method (aOR 0.82; 95% CI, 0.68–0.99) (vs. a less effective method or no method) were also lower among those diagnosed with opioid use disorder. Black enrollees were at lower adjusted odds of being provided either a moderately effective method (aOR 0.86; 95% CI, 0.84–0.89) or LARC method (aOR 0.91; 95% CI, 0.85–0.97) (vs. a less effective method or no method) compared to White enrollees. Similarly, Hispanic enrollees also were at significantly reduced adjusted odds of being provided a moderately effective method (aOR 0.69; 95% CI, 0.64–0.73) (vs. a less effective method or no method) compared to White enrollees; however, no significant difference was observed with regard to adjusted odds of LARC provision (vs. a less effective method or no method) in Hispanic versus White enrollees (aOR 1.05; 95% CI, 0.94–1.18). Having had a preventive health visit during the calendar year was associated with a four‐fold increase in adjusted odds of LARC provision (aOR 4.24; 95% CI, 4.07–4.42) (vs. a less effective method or no method) and a nearly four‐fold increase in adjusted odds of being provided a moderately effective method (aOR 3.71; 95% CI, 3.62–3.79). Finally, Kentucky Medicaid enrollees who qualified under the Affordable Care Act's Medicaid expansion were at increased adjusted odds of being provided either a moderately effective method (aOR 1.19; 95% CI, 1.16–1.21) or an LARC method (aOR 1.08; 95% CI, 1.03–1.13) (vs. a less effective method or no method) compared to those who qualified under the pre‐Affordable Care Act criteria.

### 3.4 Provider Type Differences

The provider types with the highest percentage of patients who received an LARC rather than a moderately effective method were obstetrician/gynecologists (24.5%), certified nurse midwives (23.1%), and general practitioners (20.3%) (Table 3). Notably, general practitioners were responsible for the provision of contraceptives to the largest number of enrollees in urban areas (32.0%) (Table 4). Providers in the nurse practitioner (other) category, however, were the most common providers of contraceptives in rural‐nonadjacent to urban communities, responsible for nearly a quarter of all contraceptive prescriptions (23.6%). This is particularly salient, given that less than 1 in 10 patients receiving contraceptives from providers in this category received an LARC method. Rates of LARC provision were much higher among nurse practitioners with training focused on women's health, notably certified nurse midwives (23.1%) and obstetric nurse practitioners (18.8%).

**TABLE 3:** Contraceptive type provided by prescriber specialty among Kentucky Medicaid enrollees receiving contraceptives, 2019.

Prescriber specialty^a^	Patients provided LARC, *n* (%)	Patients provided MEM, *n* (%)
General practitioner	3532 (20.3)	13,856 (79.7)
Nurse practitioner (other)	1417 (9.6)	13,419 (90.4)
Obstetrician/gynecologist	2560 (24.5)	7903 (75.5)
Family nurse practitioner	532 (6.7)	7445 (93.3)
Physician assistant	214 (8.2)	2387 (91.8)
Certified nurse midwife	707 (23.1)	2350 (76.9)
General preventive care	265 (11.1)	2114 (88.9)
Obstetric nurse practitioner	253 (18.8)	1096 (81.2)
Family practitioner	72 (6.8)	994 (93.2)
General pediatrician	81 (7.8)	958 (92.2)
Internist	15 (3.7)	392 (96.3)
Other^b^	950 (33.4)	1896 (66.6)

*Note*: Percentage values indicate row percentages.

Abbreviations: LARC, long‐acting reversible contraceptive; MEM, moderately effective method.

^a^ As reported to the Kentucky Cabinet for Health and Family Services.

^b^ Other category includes 50 provider specialties that provided contraceptives less frequently (i.e., pediatric nurse practitioner, psychiatrist, dermatologist, cardiologist, etc.).

**TABLE 4:** Prescriber of contraceptives by rurality among Kentucky Medicaid enrollees with a contraceptive provided, 2019.

Prescriber specialty^a^	Overall, *n* (%)	Urban, *n* (%)	Rural‐adjacent to urban, *n* (%)	Rural‐nonadjacent to urban, *n* (%)
General practitioner	18,945 (26.8)	11,615 (32.0)	3341 (22.7)	3989 (20.2)
Nurse practitioner (other)	15,213 (21.5)	7584 (20.9)	2985 (20.3)	4644 (23.6)
Obstetrician/gynecologist	11,771 (16.7)	6120 (16.9)	2741 (18.6)	2910 (14.8)
Family nurse practitioner	8089 (11.4)	3073 (8.5)	2016 (13.7)	3000 (15.2)
Certified nurse midwife	3176 (4.5)	1684 (4.6)	829 (5.6)	663 (3.4)
Physician assistant	2662 (3.8)	802 (2.2)	558 (3.8)	1302 (6.6)
General preventive care	2529 (3.6)	905 (2.5)	648 (4.4)	976 (5.0)
Obstetric nurse practitioner	1417 (2.0)	786 (2.2)	251 (1.7)	380 (1.9)
Family practitioner	1080 (1.5)	709 (2.0)	173 (1.2)	198 (1.0)
General pediatrician	1050 (1.5)	176 (2.1)	129 (1.2)	745 (0.7)
Internist	415 (0.6)	256 (0.7)	77 (0.5)	82 (0.4)
Other^b^	4301 (6.1)	2531 (7.0)	952 (6.5)	818 (4.2)

^a^ As reported to the Kentucky Cabinet for Health and Family Services.

^b^ Other category includes 50 provider specialties that provided contraceptives less frequently (i.e., pediatric nurse practitioner, psychiatrist, dermatologist, cardiologist, etc.).

### 3.5 Sensitivity Analyses

Sensitivity analyses showed that the addition of labor and delivery records led to an additional 708 pregnancies identified and excluded, while implementing a lookback period for LARC use (without subsequent removal) and infecund diagnoses led to an additional 15,575 individuals excluded (Appendix S2). Although these additional criteria did not impact the percentage of enrollees provided an LARC method (Appendix S3), they resulted in a modest increase in the percentage of enrollees provided a moderately effective formulation (from 22.1% to 22.9%) (Appendix S4).

## 4 Discussion

Overall contraceptive provision rates among female Kentucky Medicaid enrollees at risk of unintended pregnancy were found to be high, with estimates among those aged 15−20 corresponding well to those publicly released by the Centers for Medicaid and Medicare Services (CMS) [32]. These figures do, however, vary significantly from NSFG estimates. According to NSFG estimates from 2017 to 2019, 45.9% of female interviewees nationally aged 15−44 used a most effective or moderately effective method of contraception [33]. In our analysis, however, only 27.3% were provided a moderately effective method of contraception or an LARC. Notably, NSFG figures include other most effective methods, such as sterilization, that may, in part, explain these differences. Similar differences were observed with regard to LARC use, with 4.4% of female Kentucky Medicaid enrollees aged 15−44 provided an LARC method compared to 10.4% of those surveyed nationally in the NSFG, potentially signaling decreased LARC access among Kentucky Medicaid enrollees [33].

Additionally, our results suggest that LARC access is significantly lower among those living in rural‐nonadjacent to urban areas. Notably, these results differ from prior Oregon work, which found no significant differences in LARC utilization by rural−urban classification among Oregon Medicaid enrollees [17]. These differences highlight the variability in Medicaid populations and the healthcare landscape across US states. While our results indicate lower adjusted odds of LARC provision among those in rural‐nonadjacent to urban areas, adjusted odds of provision of a moderately effective method were found to be significantly higher among this population, as well as those living in rural‐adjacent to urban areas. This finding is supported by multiple NSFG‐based studies, which have indicated higher use of most effective or moderately effective contraceptives among those living in rural areas [14, 34]. Taken together, targeted efforts aimed at increasing LARC awareness and provision among these populations are necessary.

Our findings also suggest a positive correlation between income and contraceptive access, with those eligible for expanded Medicaid being at increased odds of both LARC and moderately effective method provision. These results are supported by prior work conducted using data from the NSFG, indicating increased contraceptive usage among those with higher incomes [35].

Our results highlight the need for further exploration in several areas, most notably provider access in rural‐nonadjacent communities, as this relates to the availability of LARC services. While providers in the nurse practitioner (other) category were the most common providers in rural‐nonadjacent communities, they provided much fewer LARCs than their physician counterparts, signaling a potential gap in training. Similar figures were observed for the family nurse practitioner category; however, obstetric nurse practitioners provided LARCs at a rate consistent with physicians. While little current data are available, a nationally representative 2009 survey noted this training gap, with only 12% of primary care nurse practitioners reporting that they were comfortable inserting an intrauterine device, compared to 72% of women's health nurse practitioners [36]. As others have described, training in intrauterine device insertion is not common outside of programs with a women's health focus [17]. Our data suggest that these training gaps among nonphysician providers may be disproportionately impacting those in rural‐nonadjacent to urban communities, resulting in lower LARC access, and thus, more universal provider training in LARC insertion may result in more equitable access to LARCs among this population.

In addition to barriers associated with provider training, other clinic‐level barriers to contraceptive access may disproportionately impact those in rural settings. A study by Okwori et al. detailed geographic differences in contraception provision and utilization among family planning clinics in South Carolina and Alabama, noting that rural clinics had less staff and were less likely to offer weekend hours or be located nearby to public transportation compared to urban clinics [37]. Taken together with our results, contraceptive access in rural areas remains complex and likely dependent on a multitude of complicating factors.

This study has several limitations to discuss. Notably, this study was conducted using data from the Kentucky Medicaid program, and thus, results are not necessarily generalizable to other populations; however, given the consistency of several of our measures with CMS figures, we believe that our results may inform future work seeking to examine contraceptive use in various populations. While our denominator was termed as those “at risk of unintended pregnancy” to align with the reporting of US contraceptive care quality measures, the data do not encompass individuals’ pregnancy desires or personal preferences; thus, many included in this study may desire pregnancy or may not be sexually active. As such, contraception provision rates should be interpreted in context with results from surveys, which can more accurately shed light on the personal characteristics than influence contraceptive use, such as marital status, sexual activity, and pregnancy intentions. Additionally, our data did not include information relative to sterilization procedures prior to the study period, and this was not defined as an outcome in our study. Given our findings relative to contraceptive access, future studies examining sterilization rates by rurality among this population are indicated. Finally, these data are from the calendar year 2019 and may not be completely reflective of more recent trends. Utilizing 2019 data, however, allowed for a better assessment of the Kentucky Medicaid population prior to the large influx of patients during the COVID‐19 pandemic. With the Kentucky Medicaid public health emergency unwinding period concluding mid‐2024, this 2019 cohort marks the last full calendar year of data without COVID‐impacted enrollment [38].

In this cross‐sectional administrative claims study, we found that, despite high moderately effective contraceptive provision among Kentucky Medicaid enrollees in rural‐nonadjacent to urban counties, odds of LARC provision are significantly lower among this population, signaling significant barriers to access and indicating that targeted efforts to increase LARC access among this population are necessary. Given provider specialty differences observed, targeted training regarding LARC provision, particularly for nonphysician providers in rural‐nonadjacent to urban areas, may mitigate access barriers and improve LARC availability.

## Funding

The project described was supported by the National Institutes of Health (NIH) National Center for Advancing Translational Sciences through grant number TL1TR001997. The content is solely the responsibility of the authors and does not necessarily represent the official views of the NIH.

## Conflicts of Interest

Dustin K. Miracle is a member of the Advisory Board of the nonprofit organization, All‐Access Eastern Kentucky, who had no input or knowledge of this work. Other authors declare no conflicts of interest or financial relationships.

## Supporting information




**Supporting File 1**: jrh70160‐sup‐0001‐SuppMat.docx

**Supporting File 2**:jrh70160‐sup‐0002‐SuppMat.docx

**Supporting File 3**:jrh70160‐sup‐0003‐SuppMat.docx

**Supporting File 4**:jrh70160‐sup‐0004‐SuppMat.docx
