# Supplementary material for: Contraceptive Method Provision Patterns Among Rural and Urban Kentucky Medicaid Enrollees
Source: J Rural Health. 2026 May 4;42:e70160. doi: 10.1111/jrh.70160 (PMC13137392; doi:10.1111/jrh.70160)
Supplement: Supplementary file 1 — Supporting File 1: jrh70160‐sup‐0001‐SuppMat.docx [file JRH-42-0-s004.docx]

## **Appendix 1.** Code List

| **Description** | **ICD-10** | **CPT/HCPCS** |
| --- | --- | --- |
| Opioid Use Disorder | F111.XXX | 99201, 99202, 99203, 99204, 99205, 99212, 99213, 99214, 99215, 99281, 99282, 99283, 99284, 99285, 99291 99304, 99305, 99306, 99307, 99308, 99309, 99310, 99324, 99325, 99326, 99327, 99328, 99334, 99335, 99336, 99337, 99341, 99342, 99343, 99344, 99345, 99347, 99348, 99349, 99350, G0402, G0438, G0439 |
| Preventive Health Visit | Z01.411, Z01.419, Z01.42, Z12.4, Z00.00, Z02.89, Z02.1, Z02.3, Z02.89, Z00.8, Z00.5 |  |
| Removal of Long-Acting Reversible Contraceptive | Z30.432 | 58301, 11982 |
