# Supplementary material for: Contraceptive Method Provision Patterns Among Rural and Urban Kentucky Medicaid Enrollees
Source: J Rural Health. 2026 May 4;42:e70160. doi: 10.1111/jrh.70160 (PMC13137392; doi:10.1111/jrh.70160)
Supplement: Supplementary file 2 — Supporting File 2:jrh70160‐sup‐0002‐SuppMat.docx [file JRH-42-0-s001.docx]

**Appendix 2.** Impact of modified exclusion criteria on number of Kentucky Medicaid enrollees aged 15-44 at risk for unintended pregnancy, 2019

|  | **Standard Method^*^** | **Additional use of labor and delivery records**^†^ | **Additional use of labor and delivery records + lookback period for infecund diagnosis**^‡^ | **Additional use of labor and delivery records + lookback period for infecund diagnosis + lookback period of LARC use^§^** |
| --- | --- | --- | --- | --- |
| Overall | 255,443 | 254,735 | 243,793 | 239,160 |
| Age |  |  |  |  |
| 15-20 | 61,760 | 61,633 | 61,502 | 61,277 |
| 21-44 | 193,683 | 193,102 | 182,291 | 177,883 |
| Diagnosis of Opioid Use Disorder |  |  |  |  |
| No | 252,433 | 251,735 | 240,951 | 236,367 |
| Yes | 3,010 | 3,000 | 2,842 | 2,793 |
| Rural-Urban Classification |  |  |  |  |
| Urban | 134,437 | 134,040 | 128,844 | 126,188 |
| Rural-Adjacent | 50,230 | 50,101 | 47,738 | 46,856 |
| Rural-Nonadjacent | 70,766 | 70,594 | 67,207 | 66,116 |
| Preventive Healthcare Visit |  |  |  |  |
| No | 187,310 | 186,895 | 179,315 | 176,445 |
| Yes | 68,133 | 67,840 | 64,478 | 62,715 |
| Race/Ethnicity |  |  |  |  |
| Black | 31,534 | 31,421 | 30,425 | 29,797 |
| Hispanic | 6,811 | 6,792 | 6,657 | 6,530 |
| White | 189,288 | 188,765 | 179,853 | 176,366 |
| Other | 27,810 | 27,757 | 26,858 | 26,467 |
| Medicaid Qualification |  |  |  |  |
| Traditional | 117,251 | 116,969 | 112,539 | 110,887 |
| Expansion | 138,192 | 137,766 | 131,254 | 128,273 |
| LARC, long-acting reversible contraceptive.  ^*^Per the U.S. Office of Population Health’s Contraceptive Care Measure reporting guidelines for 2019  ^†^Labor and delivery records were used in addition to the ICD-10 codes specified in the Contraceptive Care Measure reporting guidelines for 2019 to identify and exclude those pregnant during the last two months of the calendar year  ^‡^While Contraceptive Care Measure reporting guidelines include no lookback period to identify and exclude individuals infecund for non-contraceptive reasons, we used a 5-year lookback period in addition to the measurement year  **^§^**While Contraceptive Care Measure reporting guidelines include no lookback period to identify and exclude individuals with previous LARC placement without subsequent removal, we used a 5-year lookback period to identify and exclude these individuals | | | | |
