# Supplementary material for: Contraceptive Method Provision Patterns Among Rural and Urban Kentucky Medicaid Enrollees
Source: J Rural Health. 2026 May 4;42:e70160. doi: 10.1111/jrh.70160 (PMC13137392; doi:10.1111/jrh.70160)
Supplement: Supplementary file 3 — Supporting File 3:jrh70160‐sup‐0003‐SuppMat.docx [file JRH-42-0-s002.docx]

**Appendix 3.** Impact of modified exclusion criteria on number of Kentucky Medicaid enrollees provided a long-acting reversible method of contraception, 2019

|  | **Standard Method,^*^**  **n (%)** | **Additional use of labor and delivery records,**^†^  **n (%)** | **Additional use of labor and delivery records + lookback period for infecund diagnosis,**^‡^  **n (%)** | **Additional use of labor and delivery records + lookback period for infecund diagnosis + lookback period for LARC use,^§^**  **n (%)** |
| --- | --- | --- | --- | --- |
| Overall | 11,202 (4.4) | 11,166 (4.4) | 10,999 (4.5) | 10,602 (4.4) |
| Age |  |  |  |  |
| 15-20 | 3,020 (4.9) | 3,014 (4.9) | 3,001 (4.9) | 2,974 (4.9) |
| 21-44 | 8,182 (4.2) | 8,152 (4.2) | 7,998 (4.4) | 7,628 (4.3) |
| Diagnosis of Opioid Use Disorder |  |  |  |  |
| No | 11,082 (4.4) | 11,046 (4.4) | 10,881 (4.5) | 10,487 (4.4) |
| Yes | 120 (4.0) | 120 (4.0) | 118 (4.2) | 115 (4.1) |
| Rural-Urban Classification |  |  |  |  |
| Urban | 6,391 (4.8) | 6,367 (4.8) | 6,279 (4.9) | 6,046 (4.8) |
| Rural-Adjacent | 2,239 (4.5) | 2,234 (4.5) | 2,199 (4.6) | 2,126 (4.5) |
| Rural-Nonadjacent | 2,572 (3.6) | 2,565 (3.6) | 2,521 (3.8) | 2,430 (3.7) |
| Preventive Healthcare Visit |  |  |  |  |
| No | 5,860 (3.1) | 5,842 (3.1) | 5,765 (3.2) | 5,595 (3.2) |
| Yes | 5,342 (7.8) | 5,324 (7.8) | 5,234 (8.1) | 5,007 (8.0) |
| Race/Ethnicity |  |  |  |  |
| Black | 1,457 (4.6) | 1,450 (4.6) | 1,428 (4.7) | 1,372 (4.6) |
| Hispanic | 408 (6.0) | 403 (5.9) | 396 (5.9) | 379 (5.8) |
| White | 8,263 (4.4) | 8,241 (4.4) | 8,110 (4.5) | 7,823 (4.4) |
| Other | 1,074 (3.9) | 1,072 (3.9) | 1,065 (4.0) | 1,028 (3.9) |
| Medicaid Qualification |  |  |  |  |
| Traditional | 5,057 (4.3) | 5,042 (4.3) | 4,977 (4.4) | 4,847 (4.4) |
| Expansion | 6,145 (4.4) | 6,124 (4.4) | 6,022 (4.6) | 5,755 (4.5) |
| LARC, long-acting reversible contraceptive.  ^*^Per the U.S. Office of Population Health’s Contraceptive Care Measure reporting guidelines for 2019  ^†^Labor and delivery records were used in addition to the ICD-10 codes specified in the Contraceptive Care Measure reporting guidelines for 2019 to identify and exclude those pregnant during the last two months of the calendar year  ^‡^While Contraceptive Care Measure reporting guidelines include no lookback period to identify and exclude individuals infecund for non-contraceptive reasons, we used a 5-year lookback period in addition to the measurement year  **^§^**While Contraceptive Care Measure reporting guidelines include no lookback period to identify and exclude individuals with previous LARC placement without subsequent removal, we used a 5-year lookback period to identify and exclude these individuals | | | | |
