# Supplementary material for: Contraceptive Method Provision Patterns Among Rural and Urban Kentucky Medicaid Enrollees
Source: J Rural Health. 2026 May 4;42:e70160. doi: 10.1111/jrh.70160 (PMC13137392; doi:10.1111/jrh.70160)
Supplement: Supplementary file 4 — Supporting File 4:jrh70160‐sup‐0004‐SuppMat.docx [file JRH-42-0-s003.docx]

**Appendix 4.** Impact of modified exclusion criteria on factors associated with provision of a moderately effective method of contraception among Kentucky Medicaid enrollees, 2019

|  | **Standard Method,^*^**  **n (%)** | **Additional use of labor and delivery records,**^†^  **n (%)** | **Additional use of labor and delivery records + lookback period for infecund diagnosis,**^‡^  **n (%)** | **Additional use of labor and delivery records + lookback period for infecund diagnosis + lookback period for LARC use,^§^**  **n (%)** |
| --- | --- | --- | --- | --- |
| Overall | 56,374 (22.1) | 56,188 (22.0) | 55,266 (22.7) | 54,810 (22.9) |
| Age |  |  |  |  |
| 15-20 | 21,559 (34.9) | 21,496 (34.9) | 21,436 (34.9) | 21,386 (34.9) |
| 21-44 | 34,815 (18.0) | 34,622 (17.9) | 33,830 (18.6) | 33,424 (18.8) |
| Diagnosis of Opioid Use Disorder |  |  |  |  |
| No | 55,988 (22.2) | 55,736 (22.1) | 54,890 (22.8) | 54,439 (23.0) |
| Yes | 386 (12.8) | 382 (12.7) | 376 (13.2) | 371 (13.3) |
| Rural-Urban Classification |  |  |  |  |
| Urban | 28,198 (21.0) | 28,069 (20.9) | 27,643 (21.5) | 27,368 (21.7) |
| Rural-Adjacent | 11,733 (23.4) | 11,681 (23.3) | 11,528 (24.1) | 11,447 (24.4) |
| Rural-Nonadjacent | 16,443 (23.2) | 16,368 (23.2) | 16,095 (23.9) | 15,995 (24.2) |
| Preventive Healthcare Visit |  |  |  |  |
| No | 33,404 (17.8) | 33,254 (17.8) | 32,855 (18.3) | 32,639 (18.5) |
| Yes | 22,970 (33.7) | 22,864 (33.7) | 22,411 (34.8) | 22,171 (35.4) |
| Race/Ethnicity |  |  |  |  |
| Black | 6,484 (20.6) | 6,446 (20.5) | 6,344 (20.9) | 6,258 (21.0) |
| Hispanic | 1,358 (19.9) | 1,352 (19.9) | 1,331 (20.0) | 1,319 (20.2) |
| White | 42,710 (22.6) | 42,523 (22.5) | 41,870 (23.3) | 41,543 (23.6) |
| Other | 5,822 (20.9) | 5,797 (20.9) | 5,721 (21.3) | 5,690 (21.5) |
| Medicaid Qualification |  |  |  |  |
| Traditional | 27,489 (23.4) | 27,387 (23.4) | 27,062 (24.0) | 26,906 (24.3) |
| Expansion | 28,885 (20.9) | 28,731 (20.9) | 28,204 (21.5) | 27,904 (21.8) |
| LARC, long-acting reversible contraceptive.  ^*^Per the U.S. Office of Population Health’s Contraceptive Care Measure reporting guidelines for 2019  ^†^Labor and delivery records were used in addition to the ICD-10 codes specified in the Contraceptive Care Measure reporting guidelines for 2019 to identify and exclude those pregnant during the last two months of the calendar year  ^‡^While Contraceptive Care Measure reporting guidelines include no lookback period to identify and exclude individuals infecund for non-contraceptive reasons, we used a 5-year lookback period in addition to the measurement year  **^§^**While Contraceptive Care Measure reporting guidelines include no lookback period to identify and exclude individuals with previous LARC placement without subsequent removal, we used a 5-year lookback period to identify and exclude these individuals | | | | |
